# Clinical outcomes of the tunica albuginea plication for patients with Peyronie’s disease: a bicentric retrospective analysis

**DOI:** 10.1186/s12610-025-00264-3

**Published:** 2025-05-08

**Authors:** Pavel Navratil, Jiri Chalupnik, Minh Nguyet Tranova, Miroslav Louda, Pavel Kriz, Pavel Navratil

**Affiliations:** 1 Department of Urology, University Hospital Hradec Kralove, Sokolska 581, Hradec Kralove, 50005 Czech Republic; 2 Faculty of Medicine in Hradec Kralove, Charles University, Hradec Kralove, Czech Republic; 3 Department of Urology, Sanus, Hradec Kralove, Czech Republic

**Keywords:** Peyronie’s disease, Tunica albuginea plication, Penile curvature, Induratio penis plastica, Maladie de La Peyronie, Tunique albuginée, Plicature du Pénis, Induratio penis plastica

## Abstract

**Background:**

Tunica albuginea plication is a common surgical treatment for Peyronie’s disease that aims to correct penile curvature and improve sexual function. The goal of this study was to evaluate patient-reported outcomes, complications, and predictors of success following plication surgery in a large cohort of patients.

**Results:**

This retrospective, bicenter study included 80 patients with a mean age of 48.6 years and a mean preoperative curvature of 49.2°. Postoperative satisfaction was reported by 67% of patients. Erectile dysfunction developed in 16%, and 13% experienced complications. Among those with preoperative penile pain, 42% continued to report pain postoperatively. Reoperation was required in 12% of cases. Statistically significant associations were observed between greater preoperative curvature and higher patient satisfaction, longer follow-up duration and erectile dysfunction, and longer surgery duration and persistent pain. Patients with complex curvature patterns had a higher risk of reoperation.

**Conclusions:**

Plication surgery is an effective and safe option for treating penile curvature in Peyronie’s disease, particularly in patients with more severe deformities. Greater curvature severity predicted higher satisfaction, while longer surgeries and follow-up were associated with increased pain and erectile dysfunction. These findings can help guide patient counseling and surgical planning.

## Résumé

**Contexte:**

La plicature de la tunique albuginée est un traitement chirurgical courant de la maladie de La Peyronie qui vise à corriger la courbure du pénis et à améliorer la fonction sexuelle. L’objectif de cette étude était d’évaluer les résultats rapportés par les patients, les complications et les facteurs prédictifs de succès après une chirurgie de plicature dans une large cohorte de patients.

**Résultats:**

Cette étude rétrospective réalisée dans deux centres a porté sur 80 patients d’un âge moyen de 48,6 ans et d’une courbure préopératoire moyenne de 49,2°. La satisfaction postopératoire a été rapportée par 67 % des patients. Une dysfonction érectile s’est développée chez 16 % des patients et 13 % ont connu des complications. Parmi ceux qui souffraient de douleurs péniennes préopératoires, 42 % ont continué à signaler la présence de douleur après l’opération. Une reprise chirurgicale a été nécessaire dans 12 % des cas. Des associations statistiquement significatives ont été observées entre une plus grande courbure préopératoire et une plus grande satisfaction des patients, une durée de suivi plus longue et une dysfonction érectile, ainsi qu’une durée chirurgicale plus longue et une persistance de la douleur. Les patients présentant des profils de courbure complexes présentaient un risque plus élevé de nouvelle opération.

**Conclusion:**

La chirurgie de plicature est une option efficace et sûre pour traiter la courbure du pénis dans la maladie de La Peyronie, en particulier chez les patients présentant des déformations plus sévères. Ces résultats peuvent contribuer à guider les conseils aux patients et la planification chirurgicale.

## Introduction

Peyronie’s disease (induratio penis plastica, IPP) is a connective tissue disorder characterized by the formation of fibrous plaques in the tunica albuginea of the penis, leading to curvature, pain, and, in some cases, erectile dysfunction. The exact etiology remains unclear, though it is believed to result from microvascular trauma and aberrant wound healing. IPP predominantly affects middle-aged men, with a prevalence estimated at 3–9% [1, 2]. The degree of penile curvature varies significantly among patients and can impair both sexual function and psychological well-being.

Tunica albuginea plication (TAP) is a variation of tunica-shortening procedures, first described by Nesbit [3], aimed at correcting penile curvature. TAP is considered the least invasive surgical option and remains widely used for the treatment of Peyronie’s disease. This approach involves plicating the tunica albuginea on the convex side of the curvature to achieve straightening without excision of tissue. TAP is typically recommended for patients with stable Peyronie’s disease and significant curvature (>30°). Compared to other shortening techniques, TAP is a less invasive option, reducing the risk of sensory changes or erectile dysfunction. However, potential drawbacks include penile shortening, recurrence of curvature, and patient dissatisfaction depending on postoperative outcomes [4–7].

This study evaluates the outcomes of patients undergoing TAP for Peyronie’s disease. Our primary objectives are to analyze postoperative complications, the extent of curvature improvement, and patient satisfaction. Additionally, we aim to correlate surgical outcomes with preoperative curvature severity. By assessing these factors, we seek to provide a comprehensive understanding of the effectiveness and patient-reported outcomes associated with TAP.

## Material and methods

### Patient population

This retrospective, bicenter cohort study included 80 adult patients who underwent tunica albuginea plication for Peyronie’s disease at two urological centers between January 1st, 2014, and December 31st, 2023. This study was approved by the Institutional Review Board of University Hospital Hradec Kralove (2206P08, approved on June 22nd, 2022). Essential demographic and clinical data were collected for each patient. These included age at surgery, curvature severity (measured in degrees), the direction of curvature (ventral, dorsal, lateral, or combined), duration of surgery, length of hospitalization, postoperative complications, need for reoperation, postoperative erectile dysfunction (ED), pain, and overall patient satisfaction. Preoperative pain was defined as penile pain experienced during the active phase of the disease. All patients were operated on during the stable phase after resolution of acute symptoms. Postoperative follow-up data were gathered through routine clinical assessments and patient-reported outcomes. No patients were lost to follow-up or excluded from postoperative assessment.

### Procedure

TAP was performed using a standardized technique by one of three experienced surgeons at the participating centers. The procedure was conducted under general anesthesia. A circumferential penile degloving incision was made to expose the tunica albuginea. The degree and direction of curvature were confirmed intraoperatively via artificial erection using intracavernosal saline injection. Plication sutures were placed on the convex side of the curvature, opposite the plaque, to achieve penile straightening. Non-absorbable polypropylene 2-0 or 3-0 sutures were used, employing a horizontal mattress technique. After satisfactory penile straightening was confirmed, the tunica closure and skin closure were performed in layers. No plaque incision or grafting was performed. A compressive penile dressing was applied postoperatively, and patients were advised to abstain from sexual activity for a minimum of six weeks. Postoperative management included analgesia and prophylactic antibiotics, and patients were followed up at regular intervals to assess pain, erectile function, recurrence of curvature, and overall satisfaction.

### association between CVH, health behavior, health factor scores and CVDStatistical analysis

All statistical analyses were performed using SPSS v.27 (IBM Corp.). Descriptive statistics were calculated for all continuous and categorical variables, including means, standard deviations (SDs), and percentages. Normality of data distribution was assessed using the Shapiro-Wilk test, and parametric or non-parametric tests were selected accordingly. Pearson’s correlation coefficients were used to assess linear associations between continuous variables, including preoperative curvature and postoperative satisfaction, surgery duration and postoperative pain, and follow-up duration and erectile dysfunction. For categorical variables, chi-square tests were employed to compare proportions, such as the reoperation rate among different curvature types. A logistic regression analysis was performed to identify independent predictors of postoperative erectile dysfunction and reoperation risk, adjusting for potential confounders such as age and comorbidities. A *p*-value of <0.05 was considered statistically significant for all analyses.

## Results

The mean age of 80 patients undergoing surgery was 48.6 years (SD: 17.4), with a mean BMI of 26.4 (SD: 3.5). The mean preoperative curvature was 49.2° (SD: 19.6), and the mean follow-up duration was 24.8 months (SD: 18.2). The median follow-up duration in our cohort was 26.0 months (IQR: 14.3). The distribution of preoperative curvature types was as follows: 35% of patients had dorsal curvature, 28% had lateral curvature, 20% had ventral curvature, and 17% had a combination of multiple curvature types (dorsal+lateral or ventral+lateral). The average hospitalization duration was 2.1 days (Table 1).

**Table 1:** Patient characteristics

Characteristic		Patient cohort (*N*=80)
Mean age ± SD [years]		48.6 ± 17.4
Mean BMI ± SD [kg/m^2^]		26.4 ± 3.5
Mean preoperative curvature ± SD [°]		49.2 ± 19.6
Preoperative pain, n (%)		27 (34)
Preoperative erectile dysfunction, n (%)		16 (20)
Mean surgery duration ± SD [minutes]		42.7 ± 9.6
Mean hospitalization duration ± SD [days]		2.1 ± 0.8
Median follow-up duration (IQR) [months]		26.0 (14.3)
Curvature types, n (%)	Dorsal	28 (35)
	Lateral	22 (28)
	Ventral	16 (20)
	Combination	14 (17)

*BMI* Body mass index

In the patient cohort of 80 individuals, the mean postoperative penile curvature was 3.7° (SD: 6.0). Complete correction (defined as residual curvature ≤10°) was achieved in 86% of patients, while the remaining 14% had mild residual curvature between 11° and 20°. 67% of patients reported postoperative satisfaction following surgery. Postoperative erectile dysfunction occurred in 16% of patients, while complications were observed in 13%. Among those who experienced preoperative pain (34%), 42% continued to have persistent postoperative pain. Additionally, 12% of patients required reoperation (Table 2).

**Table 2:** Postoperative outcomes

Characteristic		Patient cohort (*N*=80)
Postoperative satisfaction, n (%)		54 (67)
Postoperative erectile dysfunction, n (%)		13 (16)
Postoperative complications, n (%)	Clavien-Dindo I	6 (8)
	Clavien-Dindo II	3 (4)
	Clavien-Dindo IIIb	1 (1)
	Total	10 (13)
Persistent postoperative pain, n (%)		11 (42) of those with preoperative pain
Reoperation, n (%)		10 (12)

A Pearson correlation test demonstrated a statistically significant positive correlation between greater preoperative curvature severity and higher postoperative satisfaction (*r* = 0.78, *p* = 0.012), indicating that patients with less severe preoperative curvature were less satisfied with their surgical outcomes. Longer follow-up duration was associated with an increased risk of postoperative erectile dysfunction (*r* = 0.31, *p* = 0.004). Patients with a follow-up of more than 24 months had a 28% higher likelihood of developing erectile dysfunction compared to those with a shorter follow-up. Surgery duration was positively correlated with persistent postoperative pain (*r* = 0.35, *p* = 0.002). Procedures lasting more than 45 minutes were associated with a significantly higher incidence of postoperative discomfort. Preoperative curvature type was associated with reoperation rates (*p* = 0.01). Patients with combination-type curvature had a 42% greater risk of undergoing reoperation compared to those with a single-plane curvature (Table 3).

**Table 3:** Key statistical associations between preoperative and postoperative variables

**Parameter**	**Correlation** ***p*-value**	**Key Finding**
Preoperative curvature severity vs. postoperative satisfaction	*r* = 0.78 *p* = 0.012	Greater preoperative curvature was associated with higher postoperative satisfaction.
Follow-up duration vs. erectile dysfunction	*r* = 0.31 *p* = 0.004	Patients with follow-up >24 months had a 28% increased risk of erectile dysfunction.
Surgery duration vs. persistent postoperative pain	*r* = 0.35 *p* = 0.002	Procedures longer than 45 minutes were linked to a higher incidence of persistent postoperative pain.
Preoperative curvature type vs. reoperation rates	*p* = 0.01	Patients with combination-type curvature had a 42% increased risk of requiring reoperation compared to single-plane curvature.

Pearson’s correlation coefficient was used to analyze associations between continuous variables. The chi-square test was used to compare categorical variables

## Discussion

Multiple studies report high patient satisfaction after penile plication, often exceeding 80%. For example, Hudak et al. found 96% of men noted improved curvature and 95% felt their overall condition improved post-plication [8]. Gholami and Lue similarly reported a 96% satisfaction rate in their series, with 93% achieving a completely straight penis [9]. Other reports have slightly lower but still strong satisfaction: Syed et al. noted 76.2% of patients were satisfied after Nesbit surgery [10], and Geertsen et al. found 82% satisfaction with tunical plication [11]. Our study’s patient satisfaction rate of 68% is on the lower end of this spectrum. This discrepancy may be due to differences in patient populations, technique or follow-up duration. Notably, not all investigations show uniformly high satisfaction – one long-term multi-center study observed that “patient dissatisfaction was the rule” for Peyronie’s disease plication outcomes [12]. In that series, only about 50% of men felt their curvature was adequately corrected, highlighting that satisfaction can vary widely. Compared to such findings, our 68% satisfaction, while lower than most contemporary reports, still indicates the majority of patients were pleased, though there remains a substantial minority who were not fully satisfied.

It is also worth noting that the mean length of hospital stay in our cohort (2.1 days) may appear prolonged compared to high-volume centers employing same-day discharge protocols. However, in our local clinical and reimbursement setting, routine postoperative observation for 1–2 nights is standard practice. This does not reflect increased surgical morbidity but rather institutional protocol.

Most literature suggests tunica plication has a minimal negative impact on erectile function, especially in the short term. Gholami and Lue reported only 4 patients (≈3%) with worsened erectile function out of 124 followed, concluding the technique has “minimal to no detrimental effect on erectile function.” [9]. A comprehensive review noted new or worsened ED rates of 0–6% after plication, indicating that in many series virtually no patients develop severe ED [12]. Indeed, Van der Horst et al. found none of their plication patients had complete ED postoperatively​ [13]. However, certain factors can influence ED outcomes. Cayan et al. observed that de-novo ED was significantly more common with the Nesbit corporoplasty than with the 16-dot plication (*p*=0.016), and that patients with Peyronie’s disease were more prone to postoperative ED than those with congenital curvature [14]. Longer-term data also suggest ED prevalence may increase over time. For instance, a 5-year follow-up study found 47.1% of men had some degree of ED after plication​ [12]. In that cohort, Peyronie’s patients fared worse – 60.5% developed ED vs only 7.7% in congenital cases. Our study noted a 16% incidence of postoperative ED. While this is slightly lower than the preoperative rate (20%), these are not directly comparable groups. Rather than improving erectile function, TAP appears to carry a low risk of new-onset erectile dysfunction when performed in patients with preserved function. We also found that longer follow-up duration was associated with a higher rate of erectile dysfunction. However, this likely reflects age-related changes or progression of underlying comorbidities over time, rather than a direct effect of the surgical procedure itself. This suggests that while plication is generally safe regarding erectile function initially, ongoing monitoring is important, especially in older patients or those with pre-existing risk factors for ED.

The effect of plication on penile pain can be twofold: it may relieve the pain of active Peyronie’s disease, but the surgery itself can introduce new discomfort. On one hand, correction of curvature often alleviates pain that was due to the disease. In the series by Van der Horst et al., the frequency of pain during intercourse was halved after Essed–Schröder plication, indicating many patients experienced pain relief post-surgery​ [13]. On the other hand, tunical plication can cause postoperative penile pain or tenderness, typically related to the plication sutures. One study reported that 24.5% of patients had persistent pain during erection long after plication surgery​ [12]. In the literature, de novo erection pain is noted as a frequent complaint – it can affect up to 60% of patients in some series (especially early after surgery), although it usually diminishes over time. Much of this pain is attributable to suture material and technique. Patients often feel palpable knots under the skin; one study noted 50% could feel plication suture bumps (with PTFE sutures), and this was as high as 88% with polypropylene stitches [12]. Such suture nodules can be tender or painful, contributing to dissatisfaction. Our findings add that longer surgeries – presumably involving more sutures or more complex dissection – were significantly associated with more persistent postoperative pain (Pearson *r* = 0.35, *p* = 0.002). This aligns with the idea that more extensive tunical manipulation may induce greater tissue trauma or nerve irritation, leading to prolonged discomfort. To mitigate pain, some authors advocate technical refinements; for example, using finer or absorbable sutures has been shown to reduce knot-related pain. Hsieh et al. reported that with an absorbable suture plication technique, no patient experienced erectile pain or palpable knots in long-term follow-up [15]. Such findings suggest that surgical technique and materials play a crucial role in pain outcomes. In summary, while many patients become pain-free or improve after plication (especially as Peyronie’s disease exits its acute phase), a notable subset can have new onset penile pain related to the procedure. Our study underscores the importance of minimizing operative time and tissue trauma to reduce chronic pain, and patients should be counseled that mild residual pain or palpable suture bumps are possible.

The need for reoperation (typically for residual or recurrent curvature) after plication varies considerably across studies, largely depending on follow-up duration and the complexity of the deformity. For example, Gholami and Lue observed a 15% recurrence of curvature at a mean 2.6 years post-plication [9]. Similarly, a recent review of surgical series noted that roughly 2–29% of patients end up requiring a secondary intervention for recurrent curvature. In one contemporary cohort (2011–2019), only 5.2% of men needed a repeat plication to fix residual curvature, which the authors noted was within the expected range compared to other studies [12]. The severity and type of curvature are key factors influencing reoperation. Multiplanar (complex) curvatures or very severe angulations are more challenging to fully correct with a single plication procedure and have higher odds of residual bend. Our findings reflect this: patients with combination-type (multidirectional) curvature had a significantly higher risk of requiring reoperation (*p* = 0.01). This is in line with clinical experience that biplanar deformities often need more extensive correction (sometimes even grafting) to achieve long-term straightening. Interestingly, Hudak et al. reported that at short-term follow-up (~14 months), men with complex curvatures achieved equivalent subjective outcomes to those with simple curvatures using plication alone [8]. However, over a longer horizon some of those “complex” cases may be more prone to recurrence. In essence, our reoperation rate for complex cases corroborates the higher end of literature estimates, emphasizing that initial surgical success does not always guarantee permanent results in Peyronie’s disease. Patients with severe or multi-angled deformities should be informed preoperatively about the elevated chance of needing a second procedure. Employing the appropriate technique for the curvature type (for instance, adding plaque incision and grafting for very complex curvatures, or using extra plication sutures strategically) may help lower the reoperation rates in these high-risk cases. Proper patient selection and setting realistic expectations are therefore crucial: while plication is effective and durable for most men, a subset – particularly those with complex deformities – may experience curvature recurrence and seek revision surgery, as reflected in both our study and the broader literature.

This study provides a comprehensive analysis of the outcomes of plication surgery, highlighting statistically significant factors influencing patient satisfaction, postoperative pain, and erectile dysfunction. The large sample size and detailed follow-up data enhance the reliability of findings. However, limitations include its retrospective design and potential selection bias. Outcomes were based on structured interviews and clinician assessments, which may introduce recall or interpretation bias. The absence of long-term functional assessments may also affect the interpretation of erectile function outcomes. Future research should focus on prospective studies with standardized outcome measures and explore strategies to minimize postoperative complications and optimize patient satisfaction.

## Conclusion

Our findings suggest that TAP is an effective and safe option for selected patients with Peyronie’s disease. While patients with more pronounced curvatures tended to report higher satisfaction, this observation likely reflects the more noticeable functional improvement. Importantly, thorough preoperative counseling and realistic expectation management may enhance satisfaction across all curvature severities.

## Data Availability

No datasets were generated or analysed during the current study.

## Abbreviations

ED: Erectile dysfunction
IPP: Induration penis plastica, Peyronie’s disease
TAP: Tunica albuginea plication
